# Supplementary material for: Optimizing the design of spatial genomic studies
Source: Nat Commun. 2024 Jun 11;15:4987. doi: 10.1038/s41467-024-49174-4 (PMC11166654; doi:10.1038/s41467-024-49174-4)
Supplement: Supplementary file 1 — Supplementary Information [file 41467_2024_49174_MOESM1_ESM.pdf]

Supplementary Material for "Optimizing the design of spatial genomic studies"

Andrew Jones[1†], Diana Cai[2†], Didong Li[3], and Barbara E. Engelhardt[4,5]
[1]*Department of Computer Science, Princeton University, USA*
[2]*Center for Computational Mathematics, Flatiron Institute, USA*
[3]*Department of Biostatistics, University of North Carolina at Chapel Hill, USA*
[4]*Gladstone Institutes, USA*
[5]*Department of Biomedical Data Science, Stanford University, USA*

† these authors contributed equally to this work

A Appendix

A.1 Supplementary methods

A.1.1 Alternate form of IG

Consider a setting in which we are choosing a design x. Suppose our model for the data y is parameterized by θ, which we wish to perform inference on. The information gain after choosing design x is given by the reduction in differential entropy from prior to posterior:

$$\text{IG}(x) = H[p(\theta)] - H[p(\theta|y, x)], \tag{A.1}$$

where $H[p(\omega)] = -\int p(\omega)\log p(\omega) d\omega = -\mathbb{E}[\log p(\omega)]$ is the entropy of a random variable ω. Expanding the posterior in Equation A.1 using Bayes rule, we have

$$\text{IG}(d) = H[p(\theta)] - H\left[\frac{p(y|\theta, x)p(\theta)}{p(y|x)}\right].$$

Note that

$$H[p(\omega_1)p(\omega_2)] = -\mathbb{E}[\log(p(\omega_1)p(\omega_2))] = -\mathbb{E}[\log p(\omega_1)] - \mathbb{E}[\log p(\omega_2)] = H[p(\omega_1)] + H[p(\omega_2)],$$

allowing us to split up the second term:

$$\text{IG}(d) = \cancel{H[p(\theta)]} - H[p(y|\theta, x)] - \cancel{H[p(\theta)]} + H[p(y|x)].$$

This yields the alternative form of IG due to the symmetry of mutual information (MI):

$$\text{IG}(d) = H[p(y|x)] - H[p(y|\theta, x)] = \text{MI}[p(y|x); p(y|\theta, x)]. \tag{A.2}$$

This is easily extended to the setting where we're choosing the design x for the tth experimental iteration, and we have already observed data $\mathbf{Y}_{1:t-1}$. In this case, our "prior" is the posterior for θ given $\mathbf{Y}_{1:t-1}$, and the IG calculation is nearly identical. This yields

$$\text{IG}(d) = H[p(y_t|x, \mathbf{Y}_{1:t-1})] - H[p(y_t|\theta, x, \mathbf{Y}_{1:t-1})] = \text{MI}[p(y_t|x, \mathbf{Y}_{1:t-1}); p(y_t|\theta, x, \mathbf{Y}_{1:t-1})]. \tag{A.3}$$

A.1.2 EIG for GP regression

Consider the GP regression model:

$$y = f(\mathbf{x}) + \epsilon, \quad f \sim GP(0, k(\cdot,\cdot)), \quad \epsilon \sim N(0, \tau^2),$$

where $\mathbf{x} \in \mathbb{R}^p$. Suppose our design space is $\mathcal{X} \subseteq \mathbb{R}^p$, and on each iteration we choose a single design $\mathbf{x}$.

Recall the differential entropy of a D-dimensional multivariate normal distribution with mean vector $\mathbf{m}$ and covariance matrix $\mathbf{C}$:

$$H[N(\mathbf{m}, \mathbf{C})] = \frac{1}{2}\log\det(\mathbf{C}) + \frac{D}{2}(\log 2\pi + 1). \tag{A.4}$$

We can plug the entropy into the equation for the EIG (Equation A.2) to compute the EIG under the GP regression model.

On the first experimental iteration (before observing any data), we can compute the EIG using Equation A.2 as follows:

$$\begin{aligned}
\text{IG}(\mathbf{X}_1) &= H[p(\mathbf{y}_1)] - H[p(\mathbf{y}_1|f)] \\
&= H[N(\mathbf{y}_1; \mathbf{0}, \mathbf{K}_{\mathbf{X}_1} + \tau^2\mathbf{I})] - H[N(\mathbf{y}_1; \mathbf{f}_1, \tau^2\mathbf{I})] \\
&= \frac{1}{2}\log\det(\mathbf{K}_{\mathbf{X}_1} + \tau^2\mathbf{I}) + \cancel{\frac{D}{2}(1+\log(2\pi))} - \frac{1}{2}\log\det(\tau^2\mathbf{I}) - \cancel{\frac{D}{2}(1+\log(2\pi))} \\
&= \frac{1}{2}\log\det(\mathbf{K}_{\mathbf{X}_1} + \tau^2\mathbf{I}) - \frac{1}{2}\log\det(\tau^2\mathbf{I}).
\end{aligned}$$

Factoring out $\tau^2\mathbf{I}$ in the log of the first term leads us to cancel the second term as follows:

$$\begin{aligned}
\text{IG}(\mathbf{X}_1) &= \frac{1}{2}\log\det\left(\tau^2\mathbf{I}\left(\frac{1}{\tau^2}\mathbf{K}_{\mathbf{X}_1} + \mathbf{I}\right)\right) - \frac{1}{2}\log\det(\tau^2\mathbf{I}) \\
&= \frac{1}{2}\log\left\{\det\left(\tau^2\mathbf{I}\right)\det\left(\frac{1}{\tau^2}\mathbf{K}_{\mathbf{X}_1} + \mathbf{I}\right)\right\} - \frac{1}{2}\log\det(\tau^2\mathbf{I}) \\
&= \frac{1}{2}\log\det\left(\frac{1}{\tau^2}\mathbf{K}_{\mathbf{X}_1} + \mathbf{I}\right) + \cancel{\frac{1}{2}\log\det(\tau^2\mathbf{I})} - \cancel{\frac{1}{2}\log\det(\tau^2\mathbf{I})} \\
&= \frac{1}{2}\log\det\left(\frac{1}{\tau^2}\mathbf{K}_{\mathbf{X}_1} + \mathbf{I}\right).
\end{aligned}$$

On experimental iteration $t > 1$, we can compute the IG using Equation A.3:

$$\begin{aligned}
\text{IG}(\mathbf{X}_t) &= H[p(\mathbf{y}_t|\mathbf{y}_{1:t-1}, \mathbf{X}_t)] - H[p(\mathbf{y}_t|f, \mathbf{y}_{1:t-1})] \\
&= H[N(\mathbf{y}_t; \mathbf{0}, \widehat{\Sigma}(\mathbf{X}_t) + \tau^2\mathbf{I})] - H[N(\mathbf{y}_t; \mathbf{f}_t, \tau^2\mathbf{I})],
\end{aligned}$$

where $\widehat{\Sigma}(\mathbf{X})$ is the posterior covariance of the GP, given by

$$\widehat{\Sigma}(\mathbf{X}_t) = \mathbf{K}_{\mathbf{X}_t,\mathbf{X}_t} - \mathbf{K}_{\mathbf{X}_t,\mathbf{X}_{t-1}}(\mathbf{K}_{\mathbf{X}_{1:t-1},\mathbf{X}_{1:t-1}} + \tau^2\mathbf{I})^{-1}\mathbf{K}_{\mathbf{X}_{1:t-1},\mathbf{X}_t}.$$

Following a similar series of calculations, we obtain

$$\text{IG}(\mathbf{X}_t) = \frac{1}{2}\log\det\left(\frac{1}{\tau^2}\widehat{\Sigma}(\mathbf{X}_t) + \mathbf{I}\right).$$

A.1.3 Variational inference approach

In the border-finding application, we approximate the posterior $p(\theta|\mathbf{y})$ using variational inference [1], where the variational family is specified as the set of multivariate Gaussians with diagonal covariance matrices. Specifically, $q(\theta) = N_K(\theta|\boldsymbol{\eta}, \text{diag}(\boldsymbol{\psi}))$, where K is the number of model parameters, $\boldsymbol{\eta}$ is the variational mean, and $\boldsymbol{\psi}$ is the vector of variational marginal variances. We then minimize the KL divergence between the approximate posterior distribution and the posterior distribution with respect to the variational parameters, $\phi = \{\boldsymbol{\eta}, \boldsymbol{\psi}\}$. This is equivalent to maximizing the evidence lower bound (ELBO). To see, this note that the KL divergence from $q(\theta)$ to $p(\theta|\mathbf{y})$ can be split into the log evidence and the ELBO:

$$
\begin{aligned}
D_{KL}(q(\theta)\|p(\theta|\mathbf{y})) &= -\mathbb{E}_q\left[\log \frac{p(\theta|\mathbf{y})}{q(\theta)}\right] \\
&= -\mathbb{E}_q\left[\log \frac{p(\mathbf{y},\theta)}{q(\theta)p(\mathbf{y})}\right] \\
&= \log p(\mathbf{y}) - \mathbb{E}_q\left[\log \frac{p(\mathbf{y},\theta)}{q(\theta)}\right] \\
&\geq 0.
\end{aligned}
$$

The KL divergence is nonnegative, so we obtain a lower bound on the log evidence:

$$
\log p(\mathbf{y}) \geq \mathbb{E}_q\left[\log \frac{p(\mathbf{y},\theta)}{q(\theta)}\right] =: \mathcal{L}(\phi). \tag{A.5}
$$

We maximize the ELBO $\mathcal{L}(\phi)$ with respect to ϕ using stochastic variational inference [1].

A.2 Experimental details

A.2.1 Allen Brain Atlas experiment

Data preprocessing. To filter out voxels outside of the tissue, we removed spatial locations with intensity less than two. The spatial coordinates were centered to have a mean of zero, and the intensities for each gene were standardized by subtracting the mean and dividing by the standard deviation.

A.2.2 Visium prostate cancer experiment

The provided graph-based cluster labels were used to label the spots containing carcinoma cells (spots belonging to Cluster 1 were labeled as carcinoma).

A.3 Supplementary figures

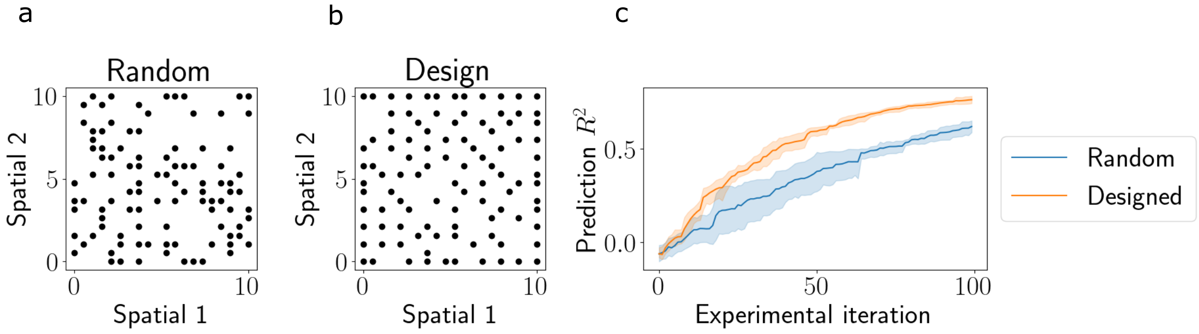

Supplementary Figure 1: **Experimental design with one-dimensional design space.** (a) Designs after $T = 100$ iterations for the *Random* approach. (b) Designs after $T = 100$ iterations for the *EIG* approach. (c) Imputation performance after each new observation. Error bands represent 95% confidence intervals computed using $n = 5$ runs.

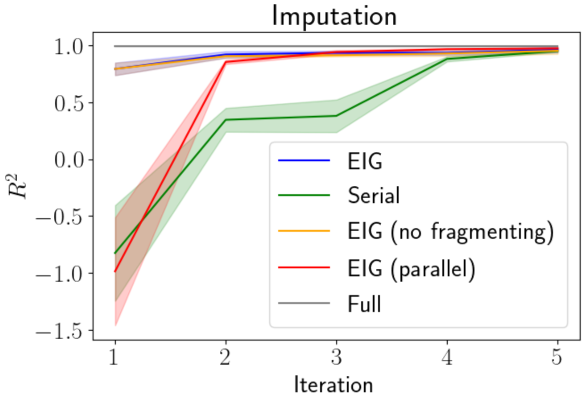

Supplementary Figure 2: **Experimental design with non-uniformly spaced cells.** This experiment follows the same protocol as the one run in Figure 2D, except the spatial locations were not sampled uniformly within a box. Instead, they were sampled from three Gaussian clusters. Error bands represent 95% confidence intervals computed using $n = 5$ runs. The numbers in this graph are captured in Supplementary Table 1.

A.4 Supplementary tables

Method	Experimental iteration	Mean	CI lower bound	CI upper bound
EIG	1	0.72	0.06	1.39
EIG	2	0.87	0.45	1.29
EIG	3	0.97	0.88	1.06
EIG	4	0.99	0.91	1.06
EIG	5	0.99	0.97	1.01
Serial	1	0.38	0.38	0.39
Serial	2	0.84	0.71	0.97
Serial	3	0.96	0.67	1.25
Serial	4	0.99	0.97	1.01
Serial	5	1.0	0.99	1.0
EIG (no fragmenting)	1	0.72	0.06	1.39
EIG (no fragmenting)	2	0.94	0.81	1.07
EIG (no fragmenting)	3	0.97	0.91	1.02
EIG (no fragmenting)	4	0.99	0.93	1.05
EIG (no fragmenting)	5	1.0	0.99	1.01
EIG (parallel)	1	0.59	-0.28	1.46
EIG (parallel)	2	0.89	0.62	1.15
EIG (parallel)	3	0.96	0.63	1.29
EIG (parallel)	4	1.0	0.99	1.0
EIG (parallel)	5	1.0	0.99	1.0
Full	1	1.0	1.0	1.0
Full	2	1.0	1.0	1.0
Full	3	1.0	1.0	1.0
Full	4	1.0	1.0	1.0
Full	5	1.0	1.0	1.0

Supplementary Table 1: Results corresponding to Fig. 2.

References

[1] Hoffman, M. D., Blei, D. M., Wang, C. & Paisley, J. Stochastic variational inference. *Journal of Machine Learning Research* (2013).
