# Supplementary material for: Optimizing the design of spatial genomic studies
Source: Nat Commun. 2024 Jun 11;15:4987. doi: 10.1038/s41467-024-49174-4 (PMC11166654; doi:10.1038/s41467-024-49174-4)
Supplement: Supplementary file 3 — Reporting Summary [file 41467_2024_49174_MOESM3_ESM.pdf]

nature portfolio

Corresponding author(s): Barbara Engelhardt

Last updated by author(s): Mar 11, 2023

Reporting Summary

Nature Portfolio wishes to improve the reproducibility of the work that we publish. This form provides structure for consistency and transparency in reporting. For further information on Nature Portfolio policies, see our Editorial Policies and the Editorial Policy Checklist.

Statistics

For all statistical analyses, confirm that the following items are present in the figure legend, table legend, main text, or Methods section.

n/a	Confirmed	
[ ]	[x]	The exact sample size (*n*) for each experimental group/condition, given as a discrete number and unit of measurement
[ ]	[x]	A statement on whether measurements were taken from distinct samples or whether the same sample was measured repeatedly
[x]	[ ]	The statistical test(s) used AND whether they are one- or two-sided<br>*Only common tests should be described solely by name; describe more complex techniques in the Methods section.*
[ ]	[x]	A description of all covariates tested
[ ]	[x]	A description of any assumptions or corrections, such as tests of normality and adjustment for multiple comparisons
[ ]	[x]	A full description of the statistical parameters including central tendency (e.g. means) or other basic estimates (e.g. regression coefficient) AND variation (e.g. standard deviation) or associated estimates of uncertainty (e.g. confidence intervals)
[x]	[ ]	For null hypothesis testing, the test statistic (e.g. *F*, *t*, *r*) with confidence intervals, effect sizes, degrees of freedom and *P* value noted<br>*Give P values as exact values whenever suitable.*
[ ]	[x]	For Bayesian analysis, information on the choice of priors and Markov chain Monte Carlo settings
[ ]	[x]	For hierarchical and complex designs, identification of the appropriate level for tests and full reporting of outcomes
[x]	[ ]	Estimates of effect sizes (e.g. Cohen's *d*, Pearson's *r*), indicating how they were calculated

Our web collection on statistics for biologists contains articles on many of the points above.

Software and code

Policy information about availability of computer code

Data collection	No software was used to collect the data.
Data analysis	Code for the model developed in this paper: https://github.com/andrewcharlesjones/spatial-experimental-design<br>Python scripts in the experiments/ directory of the above repository were used to run the experiments.<br>Other packages used:<br>numpy v1.19.2<br>pandas v1.3.5<br>scikit_learn v1.0.1<br>scipy v1.7.1<br>torch v1.8.0

For manuscripts utilizing custom algorithms or software that are central to the research but not yet described in published literature, software must be made available to editors and reviewers. We strongly encourage code deposition in a community repository (e.g. GitHub). See the Nature Portfolio guidelines for submitting code & software for further information.

Data

Policy information about availability of data

All manuscripts must include a data availability statement. This statement should provide the following information, where applicable:

- Accession codes, unique identifiers, or web links for publicly available datasets
- A description of any restrictions on data availability
- For clinical datasets or third party data, please ensure that the statement adheres to our policy

Synthetic data: Code for generating synthetic datasets can be found in the GitHub repository: https://github.com/andrewcharlesjones/spatial-experimental-design

Visium mouse brain data: Visium data were obtained from the 10x Genomics website. Data for the one slice was downloaded from the "Datasets" page. Specifically, spatial gene expression was downloaded from the page called Mouse Brain Serial Section 1 (Sagittal-Posterior) (https://www.10xgenomics.com/resources/datasets/mouse-brain-serial-section-1-sagittal-posterior-1-standard-1-1-0).

Allen Brain Atlas data: The in situ hybridization data from the Allen Brain Atlas was downloaded using the brainrender Python API. The GitHub repository for the API is available at https://github.com/brainglobe/brainrender. We used data from the study with experiment ID 79912613.

Visium prostate cancer data: Visium data were obtained from the 10x Genomics website from the Datasets page titled Human Prostate Cancer, Adenocarcinoma with Invasive Carcinoma (FFPE) (https://www.10xgenomics.com/resources/datasets/human-prostate-cancer-adenocarcinoma-with-invasive-carcinoma-ffpe-1-standard-1-3-0)

Human research participants

Policy information about studies involving human research participants and Sex and Gender in Research.

Reporting on sex and gender	Data not collected (used publicly available data only)
Population characteristics	Data not collected (used publicly available data only)
Recruitment	Used publicly available data only
Ethics oversight	Used publicly available data only

Note that full information on the approval of the study protocol must also be provided in the manuscript.

Field-specific reporting

Please select the one below that is the best fit for your research. If you are not sure, read the appropriate sections before making your selection.

[x] Life sciences [ ] Behavioural & social sciences [ ] Ecological, evolutionary & environmental sciences

For a reference copy of the document with all sections, see nature.com/documents/nr-reporting-summary-flat.pdf

Life sciences study design

All studies must disclose on these points even when the disclosure is negative.

Sample size	All data used in this study is publicly available. Full sample sizes were used based on the size of the respective available public datasets.
Data exclusions	All data used in this study is publicly available. No data were excluded from these datasets.
Replication	For all computational experiments where repetitions were possible, we included multiple randomized repetitions of the experiments to ensure their replicability. Here we performed n=5 replications across different random seeds.
Randomization	All data used in this study is publicly available. We did not perform any randomization of the available data.
Blinding	All data used in this study is publicly available. Blinding is now relevant to this study. We did not collect the data.

Reporting for specific materials, systems and methods

We require information from authors about some types of materials, experimental systems and methods used in many studies. Here, indicate whether each material, system or method listed is relevant to your study. If you are not sure if a list item applies to your research, read the appropriate section before selecting a response.

Materials & experimental systems

n/a	Involved in the study
☒	☐ Antibodies
☒	☐ Eukaryotic cell lines
☒	☐ Palaeontology and archaeology
☒	☐ Animals and other organisms
☒	☐ Clinical data
☒	☐ Dual use research of concern

Methods

n/a	Involved in the study
☒	☐ ChIP-seq
☒	☐ Flow cytometry
☒	☐ MRI-based neuroimaging
