# Supplementary figures and images for: Optimizing the design of spatial genomic studies
Source: Nat Commun. 2024 Jun 11;15:4987. doi: 10.1038/s41467-024-49174-4 (PMC11166654; doi:10.1038/s41467-024-49174-4)

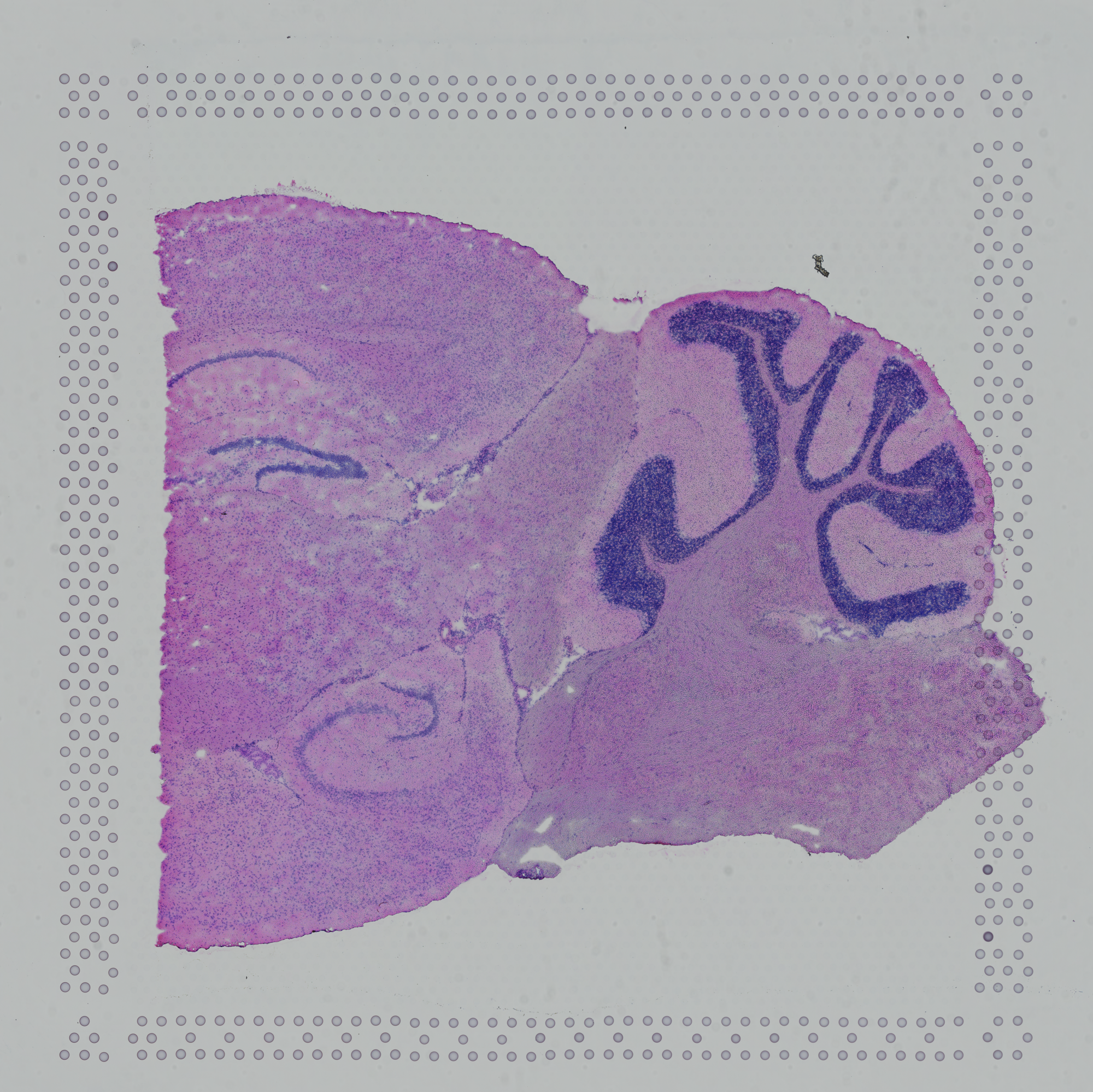

Supplement: Supplementary file 4 — Source Data [file 41467_2024_49174_MOESM4_ESM.zip › data_source_files/figure4/visium_mouse_brain/spatial/tissue_hires_image.png]

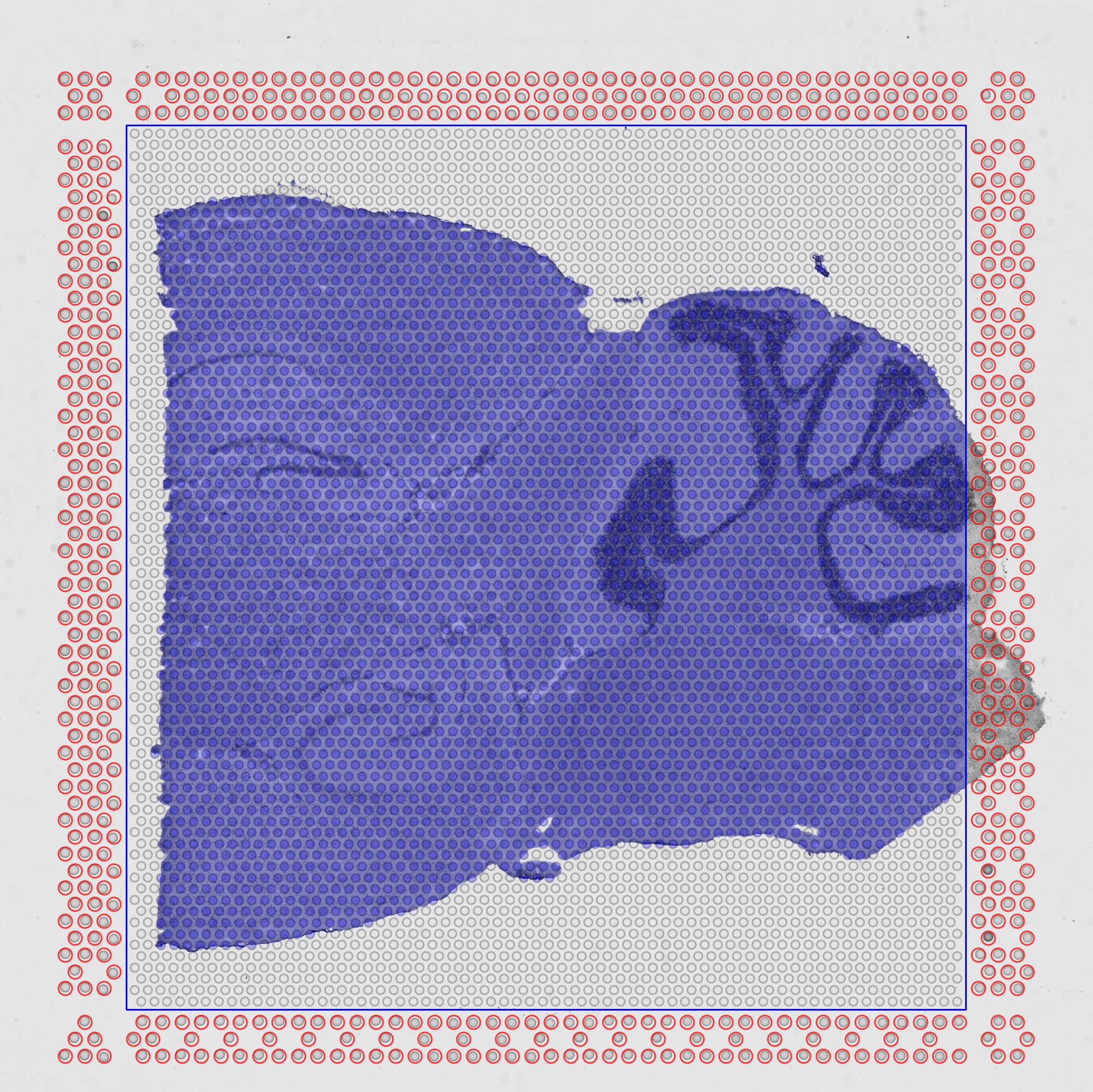

Supplement: Supplementary file 4 — Source Data [file 41467_2024_49174_MOESM4_ESM.zip › data_source_files/figure4/visium_mouse_brain/spatial/detected_tissue_image.jpg]

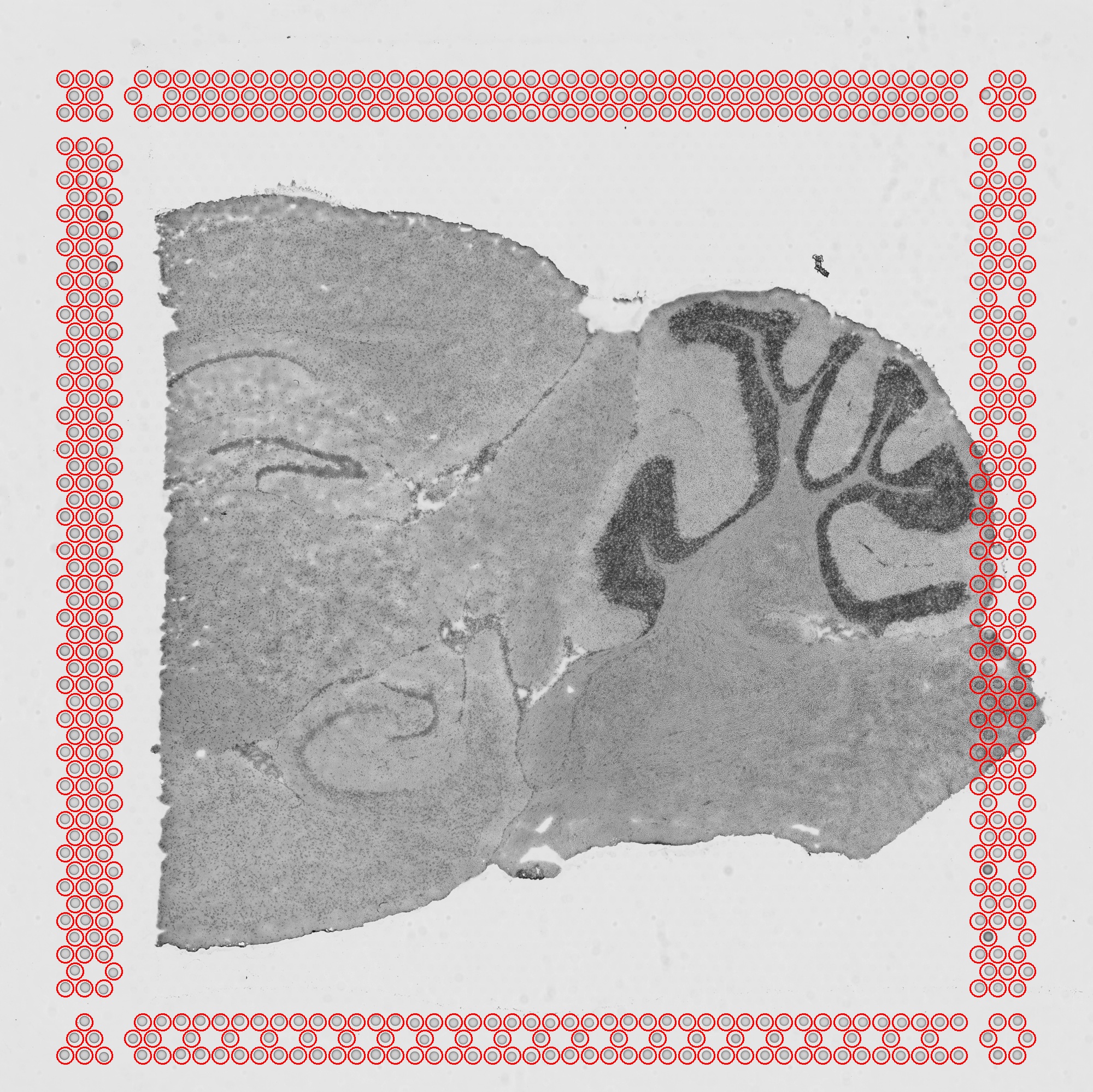

Supplement: Supplementary file 4 — Source Data [file 41467_2024_49174_MOESM4_ESM.zip › data_source_files/figure4/visium_mouse_brain/spatial/aligned_fiducials.jpg]

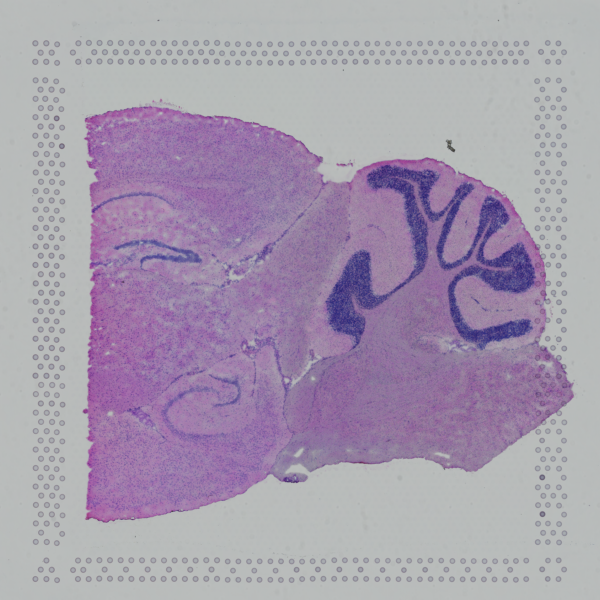

Supplement: Supplementary file 4 — Source Data [file 41467_2024_49174_MOESM4_ESM.zip › data_source_files/figure4/visium_mouse_brain/spatial/tissue_lowres_image.png]
